# Multidetector Computed Tomography (MDCT) Findings of Complications of Acute Cholecystitis. A Pictorial Essay

**DOI:** 10.3390/tomography8020095

**Published:** 2022-04-18

**Authors:** Fabio Sandomenico, Luca Sanduzzi, Emilia La Verde, Emilio Vicenzo, Luigi Pirolo, Salvatore Maione, Francesca Rosa Setola, Valeria Macchia, Umberto Dello Iacono, Domenico Barbato, Gaia Peluso, Michele Santangelo, Arturo Brunetti

**Affiliations:** 1 Radiology Unit, Buon Consiglio Fatebenefratelli Hospital, 80123 Naples, Italy; evicenzo@libero.it (E.V.); luipirolo@libero.it (L.P.); docsmaione@libero.it (S.M.); francescarosasetola@libero.it (F.R.S.); valeriamacchia@libero.it (V.M.); umberto.delloiacono.md@gmail.com (U.D.I.); 2 Diagnostic Imaging and Radiotherapy Department, Azienda Ospedaliera Universitaria “Federico II”, 80131 Naples, Italy; luca.sanduzzi@unina.it (L.S.); emilia.laverde@unina.it (E.L.V.); brunetti@unina.it (A.B.); 3 Surgery Unit, Buon Consiglio Fatebenefratelli Hospital, 80123 Naples, Italy; domenico_barbato2@virgilio.it (D.B.); gaia.peluso5@gmail.com (G.P.); santangelo.michele@fbfna.it (M.S.)

**Keywords:** abscess, aneurysm, cholangitis, colitis, computed tomography, pancreatitis, portal thrombosis, pseudoaneurysm, pylephlebitis

## Abstract

Acute cholecystitis stands out as one of the most common surgical pathologies that should always be considered in a right-upper abdominal pain emergency. For this, the importance of a correct diagnosis is well described. However, it has been demonstrated that the simple combination of clinical (pain, Murphy’s sign) and laboratory (leukocytosis) parameters alone does not provide for ruling in or ruling out the diagnosis of this condition, unless accompanied by a radiological exam. For a long time, and still today, ultrasonography (US) is by far the first-to-proceed radiologic exam to perform, thanks to its rapidity and very high sensibility and specificity for the diagnosis of simple acute cholecystitis. However, acute cholecystitis can undergo some complications that US struggles to find. In addition to that, studies suggest that multidetector computed tomography (MDCT) is superior in showing complicated forms of cholecystitis in relation to sensibility and specificity and for its capability of reformatting multiplanar (MPR) reconstructions that give a more detailed view of complications. They have shown to be useful for a precise evaluation of vascular complications, the anatomy of the biliary tree, and the extension of inflammation to surrounding structures (i.e., colitis). Therefore, based also on our experience, in patients with atypical presentation, or in cases with high suspicion for a complicated form, a MDCT abdomen scan is performed. In this review, the principal findings are listed and described to create a CT classification of acute complications based on anatomical and topographic criteria.

## 1. Introduction

### 1.1. Background

Acute cholecystitis stands out as one of the most common surgical pathologies that should always be considered in a right-upper abdominal pain emergency. For this, the importance of a correct diagnosis is well described [1]. However, it has been demonstrated that the simple combination of clinical (pain, Murphy’s sign) and laboratory (leukocytosis) parameters alone is not sufficient to rule in or out the diagnosis of this condition, and a radiologic exam is necessary [2]. For long time, and still today, ultrasonography (US) is by far the first-to-proceed radiologic exam performed, thanks to its rapidity and very high sensibility and specificity for the diagnosis of simple acute cholecystitis [3]. Nevertheless, a significant percentage of patients with acute cholecystitis admitted in the emergency department undergo complications; in some cases, patients are directly admitted with complicated acute cholecystitis as the first diagnosis. In addition to that, studies suggest that CT is superior in showing complicated forms of cholecystitis in relation to sensibility and specificity [4]. 

Wu et al. [4] concluded that the supplementary role of CT in the evaluation of complicated cholecystitis is well established, with a sensitivity rate of 88%. 

Martellotto et al. [5] reported that ultrasound was ineffective for the diagnosis of complicated acute cholecystitis in a population of 120 patients, with a sensibility of 3.5% and a specificity of 96.7%, against 34.5% and 95.6%, respectively, for CT. 

CT gives also useful surgical information, such as the severity and extension of the inflammatory process. The study also stated that, in patients who received only one examination, there were no significant differences in the diagnosis of acute cholecystitis between the two examinations. For this reason, it is likely to suppose that it could be also possible to place the surgical indication of cholecystectomy for acute cholecystitis based only on CT scan data [5]. 

The clinical diagnosis of an acute complicated cholecystitis could be challenging, especially in critically ill patients, in which the symptoms could be masked by the underlying condition. The presence of specific symptoms may be helpful for diagnosis in some cases. The occurrence of jaundice indicates an obstruction of the primary biliary duct. Severe diffuse abdominal pain is a spy of peritonitis. Hematemesis or melena is an indicative of vascular complications [5]. However, the clinical presentation could also be atypical, with only fever, nonspecific abdominal pain, and no Murphy’s sign. Therefore, based also on our experience, in patients with atypical presentation, or in cases with high suspicion of a complicated form, a contrast enhanced multidetector computed tomography (MDCT) of the abdomen is performed. Using a multidetector CT is possible to reduce scan time acquisition with thinner collimations that increase spatial resolution and lesion detection. The raw data obtained can allow the creation of multiplanar reformatted (MPR) images in the axial, coronal, or sagittal planes. MPR has proven to be essential for a precise evaluation of vascular complications, the anatomy of the biliary tree, and the precise extension of inflammation (e.g., colitis). Vascular complications, in particular, represent a specific object of interest in this review, as complete radiological information for surgical/endovascular management can only be provided by CT and not US. 

### 1.2. Objective

The purpose of this article is to describe the main CT signs of acute complications in patients suspected of complicated acute cholecystitis using the cases in our database. We aim to create a CT classification of acute complications, based on anatomical and topographic criteria, as a practical guide for the radiologist to establish the correct diagnosis and to assess proper management.

## 2. Pathophysiology

About 85%–90% of all acute cholecystitis cases are correlated with the presence of one or multiple gallstones, typically located in the neck or in the cystic duct [5]; cystic duct occlusion determines a raise in pressure within the gallbladder and ischemia of the mucosa. Bacterial infection may play a relevant role that contributes to the development and maintenance of inflammation, as well as the occurrence of complications. The condition may progress after the gallstone migrates into the main biliary duct, leading to biliary obstruction and pancreatitis, and if the flogistic insult persists, with gallbladder perforation. Acalculous cholecystitis is a far less common condition, mostly seen in patients presenting important systemic diseases (such as after major surgery, burn injury, AIDS) [6].

### 2.1. CT Findings in Acute Cholecystitis

**Figure 1:**
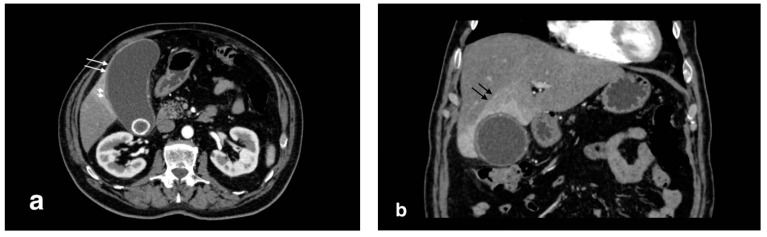
Acute uncomplicated calculous cholecystitis: (**a**) overdistended gallbladder with diffuse wall thickening and hyperemia (white arrows) and pericholecystic fluid (arrowheads), (**b**) pericholecystic parenchymal enhancement (black arrows).

Despite being less sensible than US in diagnosing acute cholecystitis, CT has proven to be essential for differential diagnosis or in patients with atypical clinical presentation. The most common features include diffuse mural thickening, gallbladder distension, pericholecystic fluid, and pericholecystic fat stranding [7] (Figure 1). A transient enhancement rim in the adjacent liver is a common finding. Despite that, CT is less sensible than US in finding gallstones, even of large dimensions in some cases [8]. In addition, mural thickening is a poorly specific criterion for diagnosis alone, as it is seen in a variety of other conditions, such as hepatitis, hypoproteinemia, heart failure, and acute pyelonephritis [9]. Acalculous cholecystitis occurs with the same CT findings, in the absence of gallstones; this form has a higher chance to develop complications (such as gangrenous cholecystitis and perforation) [6].

### 2.2. Complications

Overall, complicated acute cholecystitis has a greater risk of morbidity and mortality. Therefore, especially in critically ill patients with multiple comorbidities where the clinical presentation may vary, a tempestive radiological diagnosis is fundamental for proper treatment and correct surgical management. It has been demonstrated that age > 65 years, male gender, BMI > 25, serum leukocyte count > 10.000/serum neutrophil fraction >80%, serum platelet count < 20.000/mm3, serum ALT level > 40 IU/L, and admission via the emergency department are potential risk factors for a complicated form of cholecystitis [10], but CT remains the critical diagnostic technique to establish a definitive diagnosis. Plus, it can evidence conditions that could mimic a complicated cholecystitis, such as ulcer perforation, liver abscess, and pancreatitis [11]. For that, we aim to review the principal features, in a classification that includes complicated acute forms, gallbladder complications, biliohepatic complications, vascular complications, and extrabiliary complications.

### 2.3. Complicated Acute Forms

#### 2.3.1. Gangrenous Cholecystitis

**Figure 2:**
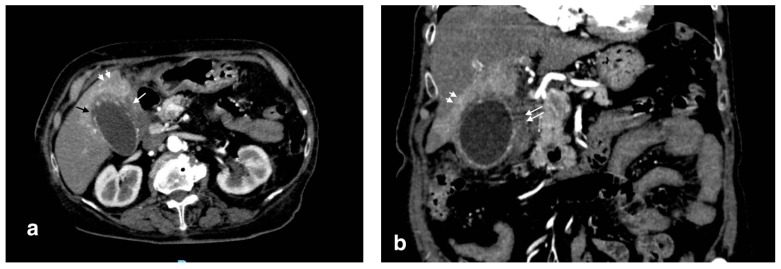
Gangrenous cholecystitis: irregular wall thickening with bulging and focal defects in the gallbladder wall (black arrows, (**a**)). Coexisting hepatic hyperemia (arrowheads) and pericholecystic fluid (white arrows) (**a**,**b**).

Gangrenous cholecystitis is a severe form of cholecystitis, characterized by transmural inflammation and wall ischemic necrosis. The risk factors include diabetes, alcohol abuse, male sex, and elevated white blood cell count [12]. The clinical presentation is similar to acute simple cholecystitis, though Murphy’s sign is more specific (100% vs. 63% in simple acute cholecystitis) [13]. The mean white blood cell (WBC) count is significantly higher and leans toward a gangrenous form against a simple form [13]. Radiologically, the presence of sloughed intraluminal membranes is characteristic. Other signs of inflammation seen in acute cholecystitis, such as wall thickening, pericholecystic fluid, and enhancement hepatic rim are often evident, as well as gallstones. Mural striations and decreased wall enhancement in a distended cholecystitis are other findings described in the literature, although not very specific [11]. A transmural defect in the gallbladder’s wall suggests perforation, a common consequence of this form (Figure 2).

#### 2.3.2. Emphysematous Cholecystitis

**Figure 3:**
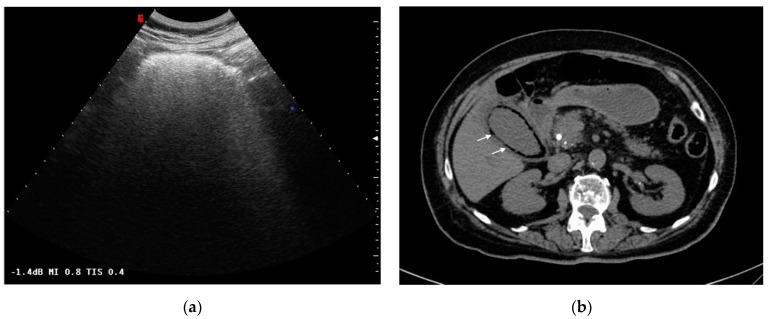
Emphysematous cholecystitis: (**a**) Ultrasonography: diffuse hyperechogenicity due to extended reverberation artifact with poor gallbladder lumen resolution. (**b**) CT findings: gallbladder wall thickening with intramural air (white arrows). CT was able to detect and characterize the disease.

This form is secondary to gallbladder infection by gas-producing organisms, such as *Clostridium* spp., in damaged gallbladder walls caused by cystic artery ischemia [7]. Thus, gallstones are not directly involved in pathogenesis. Affected patients are most commonly diabetics and mid-age males (40 to 60 years old). CT findings are similar to acute cholecystitis, but the presence of air in the gallbladder wall is characteristic and plays a key role in diagnosis (Figure 3b). Furthermore, US has a limited role in diagnosis because of the extended reverberation artifact caused by the presence of air [14] (Figure 3a).

## 3. Cholecystic Complications

### Gallbladder Perforation

**Figure 4:**
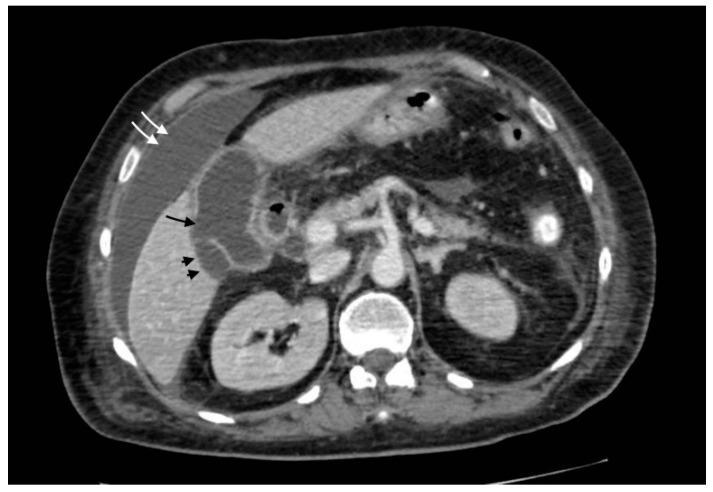
Gallbladder perforation: transmural defect of the gallbladder wall (black arrow) with pericholecystic effusion (head arrows) and perihepatic peritoneal collection, suggestive of biliary peritonitis (white arrows).

**Figure 5:**
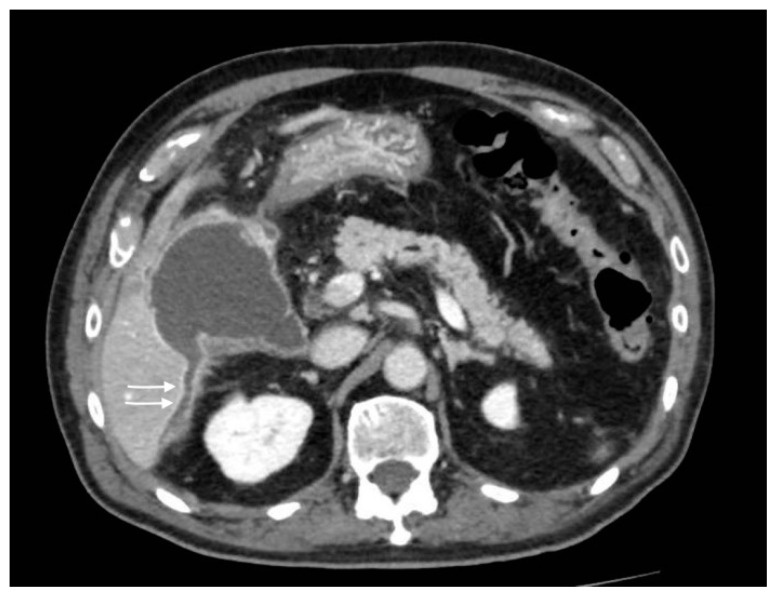
Gallbladder perforation: discontinuity of the gallbladder wall with fluid collection extended posteriorly to the right hepatic lobe (arrows).

Acute perforated cholecystitis is a severe subtype of gallbladder disease with a mortality rate up to 15%. The most known predisposing factors are infections, malignancy, trauma, drugs, and systemic diseases (heart diseases and diabetes mellitus). Although acute cholecystitis is more frequent in women, an acute perforation is more likely to be diagnosed in men. This condition is due to an extended necrosis of the gallbladder wall (more frequently at the fundus because of its relatively poor vascularization) after gallbladder distension for the presence of a gallstone obstructing the main cystic duct [15]. Clinically, the most common presentations are abdominal pain, fever, vomiting, and signs of peritonitis. Atypical presentations include abdominal wall and hepatic abscess. For the diagnosis, CT is more sensitive than US in finding a transmural defect in the gallbladder wall or extraluminal position of gallstones, two specific signs of perforation [16]. Other findings that have been described in the literature are pericholecystic fluid, abscess (or fluid collection), gallbladder wall thickening, layering gallbladder wall, gallbladder wall bulging, omental/mesenteric stranding, and peritoneal air or peritoneal fluid, suggestive of biliary peritonitis (Figure 4) [17]. The site of perforation determines the point of exit of intraluminal gallstones, bile, and the rest of the content within the gallbladder (Figure 5); therefore, the perforation might lead to pericholecystic abscess or biliary peritonitis.

**Figure 6:**
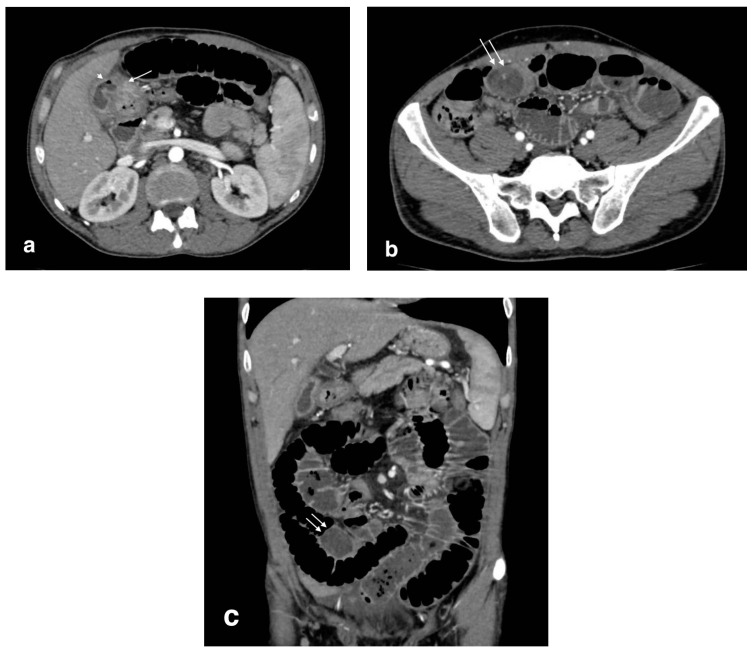
Bilioenteric fistula (biliogastric) with gallstone ileus: (**a**): cholecystogastric fistula (arrow) with the presence of air in the gallbladder lumen (arrowhead); (**b**) axial image shows a large gallstone in the ileum (arrows) with small bowel overdistension (white arrows); (**c**) coronal image demonstrates an obstructive gallstone in the ileum (arrows) with small bowel overdistension.

A pathological communication between the gallbladder and GI tract can develop in the setting of an acute cholecystitis, or after recurrent episodes, in which the formation of adherences favors the genesis of a fistula. Usually, the fistula develops between the gallbladder and the small bowel, most frequently in the duodenum, but other locations are possible, even if less common, such as in the stomach or colon [18]. MDCT scans, and in particular multiplanar reconstruction, should be performed to evaluate the continuity between the organs. *Gallstone ileus* is a rare complication in which a large gallstone (usually >2.5 cm), due to the formation of a fistula, migrates and impacts into the small bowel (most frequently in the distal ileum), leading to small bowel obstruction. CT is essential for diagnosis, through the evidence of Rigler’s triad (pneumobilia, small bowel obstruction, and large gallstone in the ileus in 79% of the patients) (Figure 6) [19]. After a gallstone is visualized within the extrabiliary GI tract, it is recommended to look for the presence of other gallstones outside the biliary system. 

## 4. Biliary and Intrahepatic Complications

### 4.1. Acute Cholangitis

**Figure 7:**
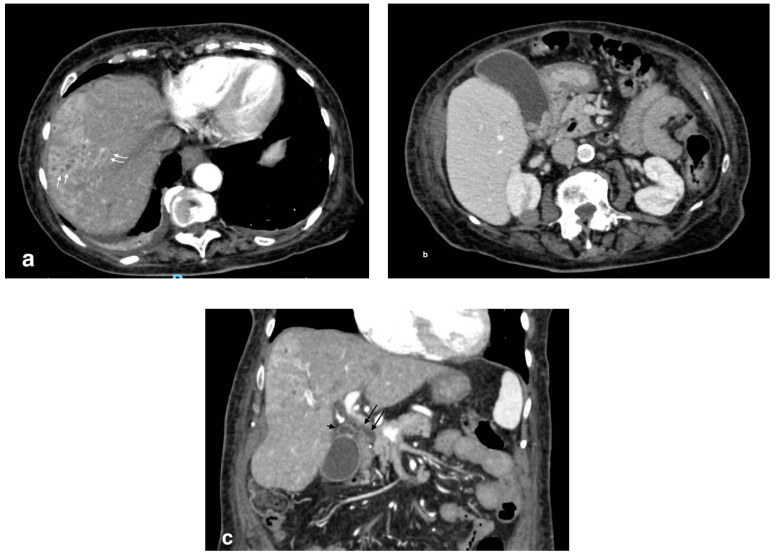
Cholangitis with microabscesses: (**a**) axial image shows patchy biliary intrahepatic duct dilation (curved arrows) and small hypodense collections due to microabscesses (white arrows); (**b**) overdistended gallbladder with infundibular stones and thickening of the gallbladder due to calculous cholecystitis; (**c**) coronal scan shows a dilated common bile duct (arrow) and cystic duct (arrowhead) with wall hyperdensity due to inflammation.

Acute cholangitis as a complication of acute cholecystitis occurs after gallstone migration and impaction into the common bile duct, determining obstruction and biliary stasis that favors overinfection. As a result, these patients manifest abdominal pain, fever, and jaundice (Charcot’s triad) [20,21]. The most important signs to support the diagnosis of cholangitis on MDCT in an emergency context are biliary tract obstruction and intra/extrahepatic bile duct dilation (Figure 7) [22]. During the arterial phase, inhomogeneous transient hepatic parenchymal enhancement appears, which could be seen as nodular, patchy, wedge-shaped, or geographic. Complications of acute cholangitis are represented by hepatic abscess, portal vein thrombosis, and biliary peritonitis [15]; MD CT supports diagnosis for these condition also.

### 4.2. Pericholecystic and Liver Abscesses

**Figure 8:**
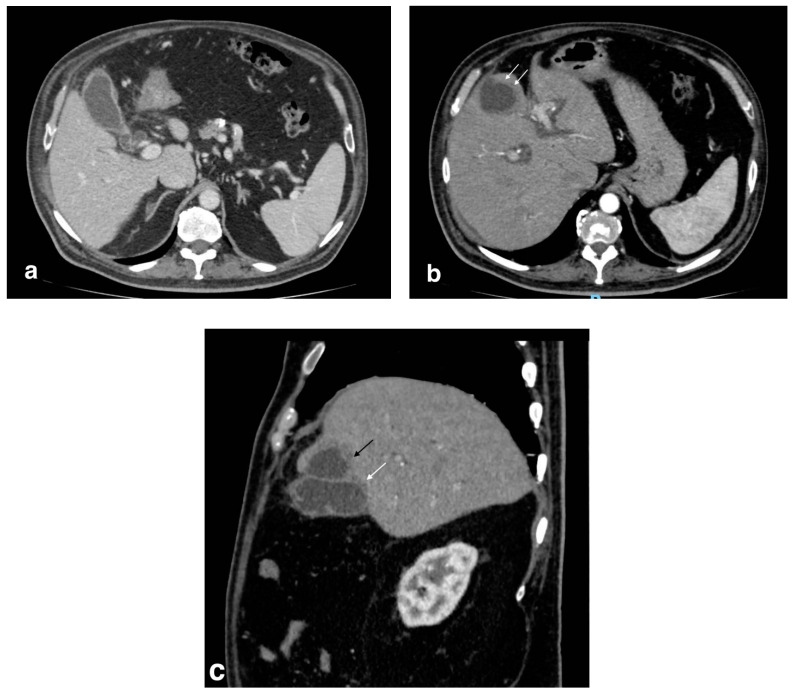
Pericholecystic abscess: (**a**) axial images show acute cholecystitis findings with wall thickening and hyperdensity; (**b**) a hypoattenuated sovracholecistic collection (white arrows) due to hepatic pericholecystic abscess; (**c**) sagittal image evidences the proximity of the two structures (abscess: black arrow; cholecyst: white arrow).

**Figure 9:**
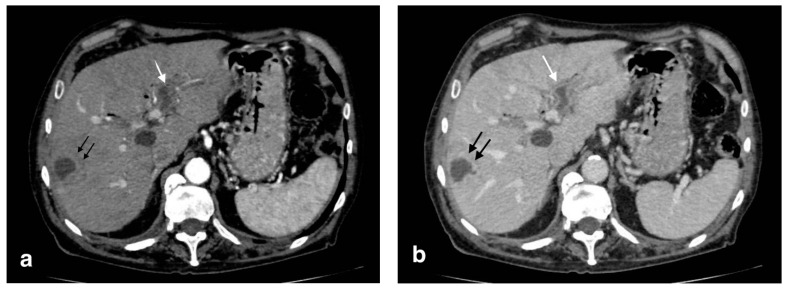
Liver abscess with pylephlebitis: (**a**) CT arterial phase; (**b**) CT portal phase. In the right lobe, V segment, a large hypodense collection with hypoattenuating halo due to intrahepatic abscess (black arrows, (**a**,**b**)) with the presence of a hypodensity within the left portal vein lumen due to thrombosis and hyperdensity of portal walls indicative of pylephlebitis better defined in CT portal phase (**b**) (white arrow).

In the context of acute cholecystitis, the formation of liver abscess is an event strictly related to a gallbladder perforation. It can be differentiated in pericholecystic and parenchymal abscesses. The leakage of bile and other material from the gallbladder lumen can undergo infection (it could also be already infected) and finally organize in a mass, sometimes multiloculated, with a rounded contour and a more or less thick capsule, which can be collected in the pericholecystic space or, distally, in the liver parenchyma. Pericholecystic abscess as a complication of acute cholecystitis is reported in 3%–19% of cases [17]. The typical presentation consists in the evidence of intramural and pericholecystic rim-enhancing fluid collections, with the omentum often adherent/thickened (Figure 8). The abscess can be unilocular or have septations and an irregular contour [17]. Liver abscesses are classified by size: macroabscesses are more than 2 cm in diameter, and microabscesses are less than 2 cm in diameter [15]. Pathogenically, liver abscesses could also originate from an ascending infection from the biliary system [20]; in this case, MDCT reveals the presence of gallstones obstructing the biliary duct and/or suppurative cholangitis. After contrast injection, peripheral contrast enhancement is often shown (the so-called “rim sign”) (Figure 9). The presence of intralesional air is almost pathognomonic, despite being not always detectable. 

## 5. Vascular Complications

### 5.1. Portal Vein Thrombosis (PVT) and Pylephlebitis

**Figure 10:**
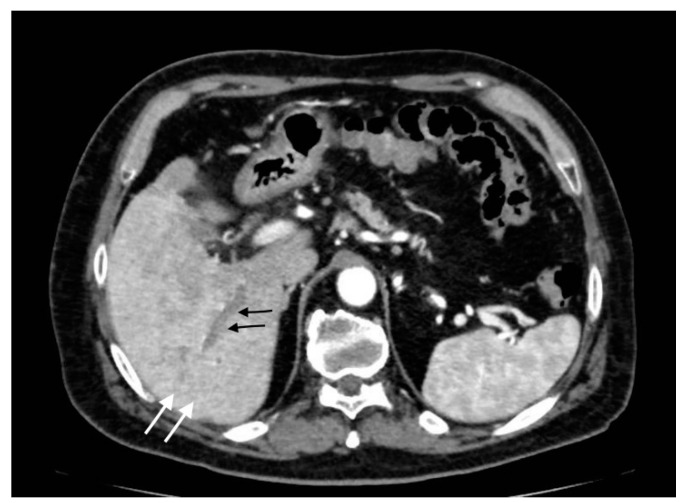
Right portal vein thrombosis: extensive right portal vein hypodensity subsequent to an intraluminal thrombus formation (black arrows) with hepatic hyperemia (white arrows).

**Figure 11:**
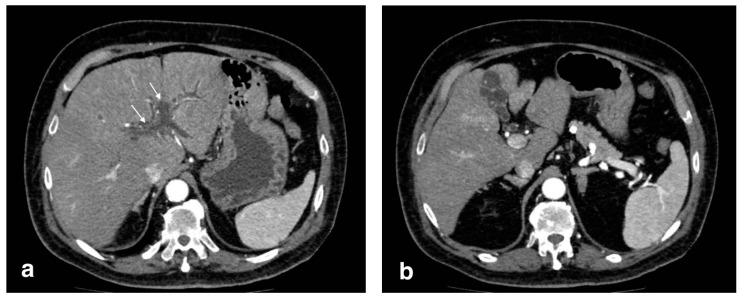
Pylephlebitis in cholecystitis exacerbation: (**a**) massive thrombosis of the portal bifurcation with hyperdensity of portal walls due to pylephlebitis (white arrows) and inhomogeneous attenuation of liver parenchyma in arterial CT scan due to vascular occlusion; (**b**) axial scan: irregular thickening of cholecystic walls with contrast enhancement of liver parenchyma due to pericholecystic edema.

Acute portal vein thrombosis is an uncommon condition caused by the formation of a thrombus within the portal vein, as the inflammatory insult persists. Besides acute cholecystitis, it could be diagnosed as a complication of many other conditions, such as diverticulitis, urinary and pelvic infections, and malignancies [23,24]. Symptoms may be subtle, often masked by the underlying condition. CT and US are the main modalities for the acute assessment of PVT. The lack of enhancement within the vein lumen and an enlarged portal vein are the two main classic imaging CT features [24,25]. CT, unlike US, is also able to evaluate the extension of the thrombus and to detect associated findings, such as hepatic hyperemia (Figure 10), or complications, such as enteric ischemia or intra-abdominal collections [24]. Suppurative thrombosis of the portal vein, also known as pylephlebitis, is a life-threatening condition that requires early diagnosis and therapeutic assessment. The exact pathogenesis is not well known, but it is proven that specific bacterial species, such as *Bacteroides Fragilis*, play a key role in favoring a protrombothic state [26] and the clinical features of sepsis. As regards PVT, CT evaluation is meaningfully superior to ultrasonography for the diagnosis. Consequent to hepatic artery overflow due to PVT occlusion, transient contrast enhancement of the adjacent hepatic parenchyma during the arterial phase may occur, as well as hepatic microabscesses (Figure 11) [27]. Although not seen in most cases, CT may detect the presence of air within the thrombus as a hypodense focus within the thrombosed PV [24]. 

### 5.2. Visceral Arteries Pseudoaneurysm

**Figure 12:**
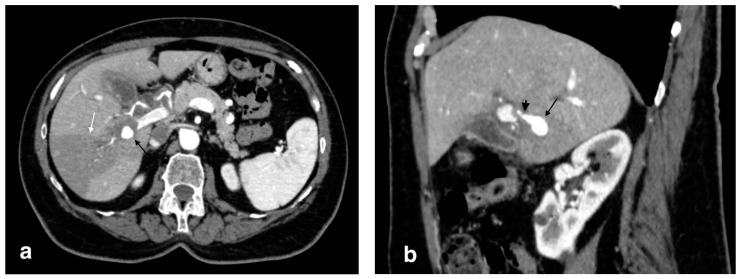
Right hepatic artery pseudoaneurysm: (**a**) axial image in the arterial phase shows an abnormal dilation of the right hepatic artery (black arrow); wedge-shaped area of parenchymal hypodensity (white arrow) secondary to hypoperfusion; (**b**) sagittal image better demonstrates continuity of the abnormal dilation (arrow) with a right hepatic artery branch (arrowhead).

Hepatic artery aneurysms are very rare, accounting for 20% of visceral splanchnic aneurysms (1% of all aneurisms). Pseudoaneurysm represents over 50% of hepatic artery aneurysms, which differ from true aneurysms because they do not involve the entire arterial wall but only a focal wall defect surrounded by fibrotic material, leading to a false layer [28]. Nevertheless, the risk of rupture is significantly higher for pseudoaneurysms [24]. It can be responsible for hemobilia [23]. The literature, and our experience, suggests that acute cholecystitis represents a potential cause of pseudoaneurysms; in fact, as reported in the literature, severe inflammation can cause adjacent arterial wall degeneration, resulting in pseudoaneurysm formation [28,29,30]. For acute cholecystitis, cystic and right hepatic arteries are generally involved [24]. CT shows this lesions as an abnormal vessel dilatation with irregular contour and sometimes surrounded by hematomas. Hepatic artery pseudoaneurysm could be seen as an artery-phase homogenously enhanced intrahepatic mass (Figure 12); however, it may also be detected extrahepatically when it originates from the common or proper hepatic arteries [24]. Diagnostic assessment is fundamental for proper treatment: recently, endovascular treatment (percutaneous embolization or stent grafting) is preferred to surgical repair [31]; however, this type of lesion may resolve spontaneously [32].

## 6. Extrabiliary Gastrointestinal Complications

### 6.1. Colitis

**Figure 13:**
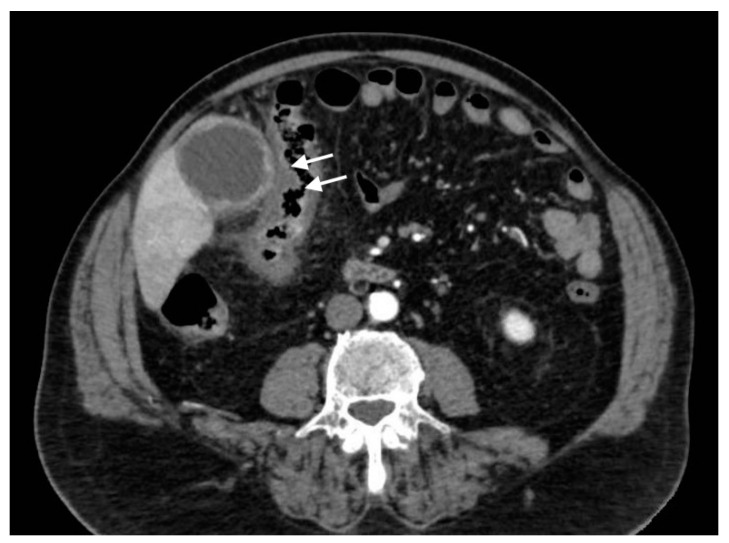
Gangrenous cholecystitis with colitis: axial scan shows the signs of pericholecystic inflammation that extend to the right colic flexure with diffuse colic wall thickening (white arrows).

Persistent gallbladder inflammation may extend to pericholecystic tissues and to the right side and hepatic flexure of the colon, causing fat stranding and thickening with a hypodensity of colic walls due to edematous infiltration (Figure 13).

### 6.2. Biliary Pancreatitis

**Figure 14:**
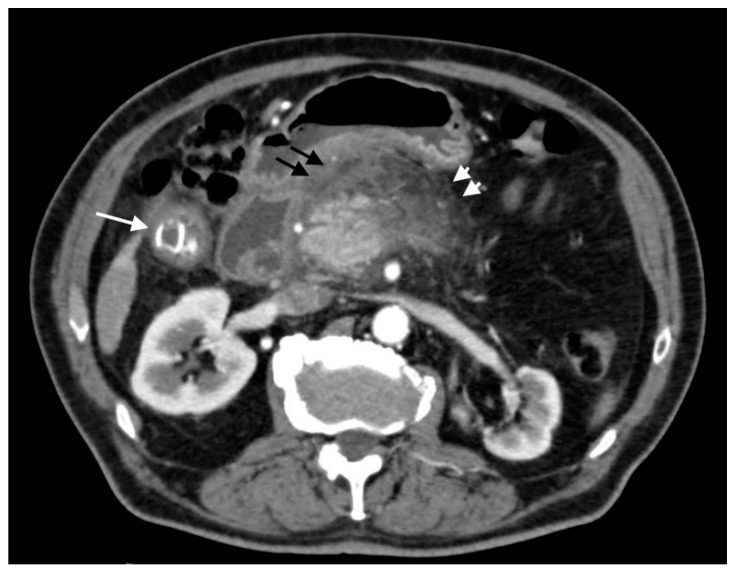
Acute calculous cholecystitis with pancreatitis (biliary pancreatitis): axial scan shows an acute cholecystitis (white arrow) with edematous hypoattenuating pancreatitis with surrounding peripancreatic fat strands (arrowheads) and fluid (black arrows).

The progression of gallstones or biliary sludge and the impaction at the ampulla of Vater lead to ampullary spasm, pancreaticobiliary reflux, and the obstruction of the common and pancreatic ducts, leading to acute biliary pancreatitis and cholangitis [15]. Findings on contrast-enhanced CT include an edematous hypoattenuating pancreas with surrounding peripancreatic inflammation and fluid associated with signs of obstructive bile duct pathology due to gallstones (Figure 14). Severe cases may include findings of pseudocyst formation or pancreatic necrosis [15].

## 7. Conclusions

Contrast-enhanced CT should be performed if a complicated form of acute cholecystitis is suspected. In fact, despite being utilized for the diagnosis of acute cholecystitis for its sensibility and rapidity, US does not always provide useful information about its complications, in which the diagnosis remains uncertain. Furthermore, CT plays an essential role in evaluating the extension and the severity of the condition and in surgical planning.

## Data Availability

The data presented in this study are available on request from the corresponding author. The data are not publicly available due to privacy.
